# Construction of a novel nomogram based on competing endogenous RNAs and tumor-infiltrating immune cells for prognosis prediction in elderly patients with colorectal cancer

**DOI:** 10.1007/s12672-023-00742-y

**Published:** 2023-07-10

**Authors:** Zhimin Tao, Bo Li, Chunyan Kang, Wei Wang, Xianzhe Li, Yaowu Du

**Affiliations:** 1 School of Nursing and Health Sciences, Henan University, Kaifeng, 475000 Henan China; 2 Henan Medical College, Kaifeng, 475000 Henan China; 3 The First Affiliated Hospital of Henan University, Kaifeng, 475000 Henan China; 4 Division of Clinical Epidemiology and Aging Research, German Cancer Research Center (DKFZ), 69120 Heidelberg, Germany; 5 Medical Faculty Heidelberg, Heidelberg University, 69120 Heidelberg, Germany; 6 School of Basic Medical Sciences, Henan University, Kaifeng, 475000 Henan China

**Keywords:** Competing endogenous RNAs, Immune cell, Nomogram, Prognosis, Elderly patient, Colorectal cancer

## Abstract

Competitive endogenous RNAs (ceRNAs) and tumor-infiltrating immune cells play essential roles in colorectal cancer (CRC) tumorigenesis. However, their prognostic role in elderly patients with CRC is unclear. Gene expression profiles and clinical information for elderly patients with CRC were downloaded from The Cancer Genome Atlas. Univariate, LASSO, and multivariate Cox regression analyses were utilized for screening key ceRNAs and prevent overfitting. A total of 265 elderly patients with CRC were included. We constructed a novel ceRNA network consisting of 17 lncRNAs, 35 miRNAs, and 5 mRNAs. We established three prognosis predictive nomograms based on four key ceRNAs (ceRNA nomogram), five key immune cells (immune cell nomogram), and their combination (ceRNA-immune cell nomogram). Among them, the ceRNA-immune cell nomogram had the best accuracy. Furthermore, the areas under the curve of the ceRNA-immune cell nomogram were also significantly greater than the TNM stage at 1 (0.818 vs. 0.693), 3 (0.865 vs. 0.674), and 5 (0.832 vs. 0.627) years. Co-expression analysis revealed that CBX6 was positively correlated with activated dendritic cells (R = 0.45, p < 0.01), whereas negatively correlated with activated mast cells (R =− 0.43, p < 0.01). In conclusion, our study constructed three nomograms to predict prognosis in elderly patients with CRC, among which the ceRNA-immune cell nomogram had the best prediction accuracy. We inferred that the mechanism underlying the regulation of activated dendritic cells and mast cells by CBX6 might play a crucial role in tumor development and prognosis of elderly patients with CRC.

**Supplementary Information:**

The online version contains supplementary material available at 10.1007/s12672-023-00742-y.

## Introduction

Colorectal cancer (CRC) is a prevalent gastrointestinal malignancy worldwide [1]. The highest percentages of morbidity and mortality of CRC mainly pertain to elderly patients ( ≥  65 years old), with nearly 70% of cases diagnosed in those older than age 65 [2, 3]. Compared to young patients with CRC, elderly patients exhibit several key differences. Firstly, the incidence of colorectal cancer increases with age, making it more prevalent among the elderly population [4]. Additionally, elderly patients often present with different clinical features, including a greater proportion of advanced-stage disease at diagnosis, and a higher prevalence of comorbidities [5]. Last but not least, elderly patients with CRC undergoing surgery are more likely to have a poor prognosis and survival rates than younger patients [6, 7]. Thus, it is necessary to identify new prognostic markers and explore the potential mechanism of poor prognosis in elderly patients with CRC, thereby improving their survival and leading to better management and treatment of the disease.

In 2011, the hypothesis of competitive endogenous RNA (ceRNA), proposed by Salmena et al., held that all types of RNA transcripts, such as lncRNAs, mRNAs, and other RNAs, can act as ceRNAs via microRNA response elements that bind to miRNAs [8]. Based on this hypothesis, an increasing number of studies further confirmed the significance of the lncRNA-miRNA-mRNA regulatory network in the initiation and progression of various tumors. For example, Wang et al. [9] elucidated that cAMP-responsive element-binding protein regulates the expression of HULC by competitively binding endogenous miR-372 in liver cancer. In addition, Wang et al. [10] found a novel ceRNA regulatory network in gallbladder cancer. The ceRNA network has also been suggested as a promising prognosis biomarker in cancer, including pancreatic cancer, melanoma, and other tumors [11, 12].

The tumor microenvironment is a complex network containing many cells, such as tumor, mesenchymal, and infiltrating immune cells [13]. To date, numerous studies have revealed the crucial role of tumor and tumor-infiltrating immune cells in the progression of cancer [14] and their tight association with prognosis [15, 16]. The communication between tumor and tumor-infiltrating immune cells is often regulated by the ceRNA network [8]. Some studies have recently demonstrated that the ceRNA network and tumor-infiltrating immune cells have an excellent predictive value for the prognosis of soft tissue sarcoma and mesothelioma [17, 18]. Therefore, it is essential to understand the role of the ceRNA network and tumor-infiltrating immune cells in elderly patients with CRC.

This study aimed to construct and validate nomograms based on the ceRNA network and tumor-infiltrating immune cells for prognosis prediction in elderly patients with CRC. We constructed a ceRNA network based on the different gene expression profiling and calculated the proportions of immune cells between normal and tumor samples in elderly patients with CRC. Accordingly, three nomograms based on key ceRNAs, key immune cells, or their combination were established to predict the 1-, 3-, and 5-year overall survival (OS) in elderly patients with CRC. We used the area under the curve (AUC) of the receiver operating characteristic (ROC) analysis, concordance index (C-index), and calibration curves to assess the accuracy, discrimination, and calibration of these three nomograms. In addition, a correlation analysis of key ceRNAs and immune cells was performed to explore the potential mechanism underlying poor prognosis in elderly patients with CRC.

## Methods

### Data acquisition

We downloaded the gene expression profiling data of 312 samples from elderly patients with CRC (aged ≥ 65) with complete clinical data (including 252 tumor and 60 adjacent normal samples) from The Cancer Genome Atlas (TCGA) (https://tcga-data.nci.nih.gov/tcga/). After filtering out genes that were not differentially expressed between tumor and normal samples, differential expression analysis of mRNAs, lncRNAs, and miRNAs was performed using the “DESeq2” package in R software. The threshold was set at false discovery rate (FDR) < 0.05 and |log2fold change (FC)| > 1.

### Construction of the ceRNA network

To improve prediction accuracy, we downloaded experimentally verified information on miRNA-mRNA and lncRNA-miRNA interactions from the starBase database (http://starbase.sysu.edu.cn/) [19]. Subsequently, the eligible lncRNAs, miRNAs, and mRNAs were chosen based on hypergeometric detection and correlation analysis results with a significance threshold of p < 0.05. These selected molecules were used to construct the ceRNA network, which was visualized using Cytoscape v.3.5.1 software [20].

### Survival analysis and nomogram of key ceRNAs

The prognostic value of all members in the ceRNA network was assessed using Kaplan-Meier survival analysis and the Cox proportional hazard model. LASSO Cox regression was employed to identify and eliminate potential overfitting factors within the ceRNA network. Subsequently, based on the expression levels and coefficients of each feature in the Cox model, a risk score was calculated for elderly patients with CRC as follows:

$$risk \,score = \sum _{i=1}^{k}\beta iSi,$$

where k is the number of key genes included in the ceRNA network,$\beta i$ is the coefficient per gene, and $Si$ is the level of gene expression [21, 22]. Patients were categorized into high- and low-risk groups based on the median risk score as the cut-off value. Subsequently, survival analysis was conducted between the high-risk group and low-risk group. We then established a nomogram based on the Cox proportional hazard model to predict the survival rate of elderly patients with CRC. The accuracy, discrimination, and calibration of the nomogram in predicting the 1-, 3-, and 5-year OS were evaluated using the C-index, AUC of ROC analysis, and calibration curves.

### Immune landscape assessment

To further explore the relationship between the onset of colorectal cancer and the tumor microenvironment in elderly patients with CRC, the CIBERSORT algorithm was employed to estimate the relative abundance of 22 immune cells in CRC samples obtained from elderly patients. Samples with a p-value less than 0.05 were defined statistically significant and could be included in Wilcoxon rank-sum tests between normal and tumor samples.

### Survival analysis and nomogram of key immune cells

We identified immune cell types associated with prognosis using Kaplan-Meier survival and Cox regression analyses. LASSO Cox regression was applied to prevent the overfitting of immune cells. Similar to the method previously described, the risk score of each patient was calculated according to the coefficient and expression levels of each immune cell in the Cox model, followed by the classification of patients into high- and low-risk groups based on their median risk score. We then selected the immune cells in the model to establish a nomogram. The prediction efficiency of the nomogram was reflected through the C-index, ROC analysis, and calibration curves. Finally, to explore whether combining key ceRNAs and immune cells results in improved prediction accuracy, we constructed a combined nomogram and verified its accuracy, discrimination, and calibration using the same methodology.

### External validation of the protein expression level corresponding to mRNAs

To validate the accuracy of the results from the bioinformatics analysis and reduce cohort bias, we detected the levels of protein expression corresponding to key mRNAs in the ceRNA signature at the tissue level using the Human Protein Atlas database [23].

### Statistical analysis

The R version 4.0.3 was used for all statistical analyses (packages: GDCRNATools, DESeq2, ggplot2, RMS, glmnet, survminer, and timeROC). The Wilcoxon test was employed to compare two independent nonparametric samples. The chi-square test was utilized for comparing categorical variables. Correlations between variables were analyzed using Spearman’s correlation coefficient. A *p* < 0.05 was considered statistically significant.

## Results

### Identification of significant differentially expressed genes

**Fig. 1:**
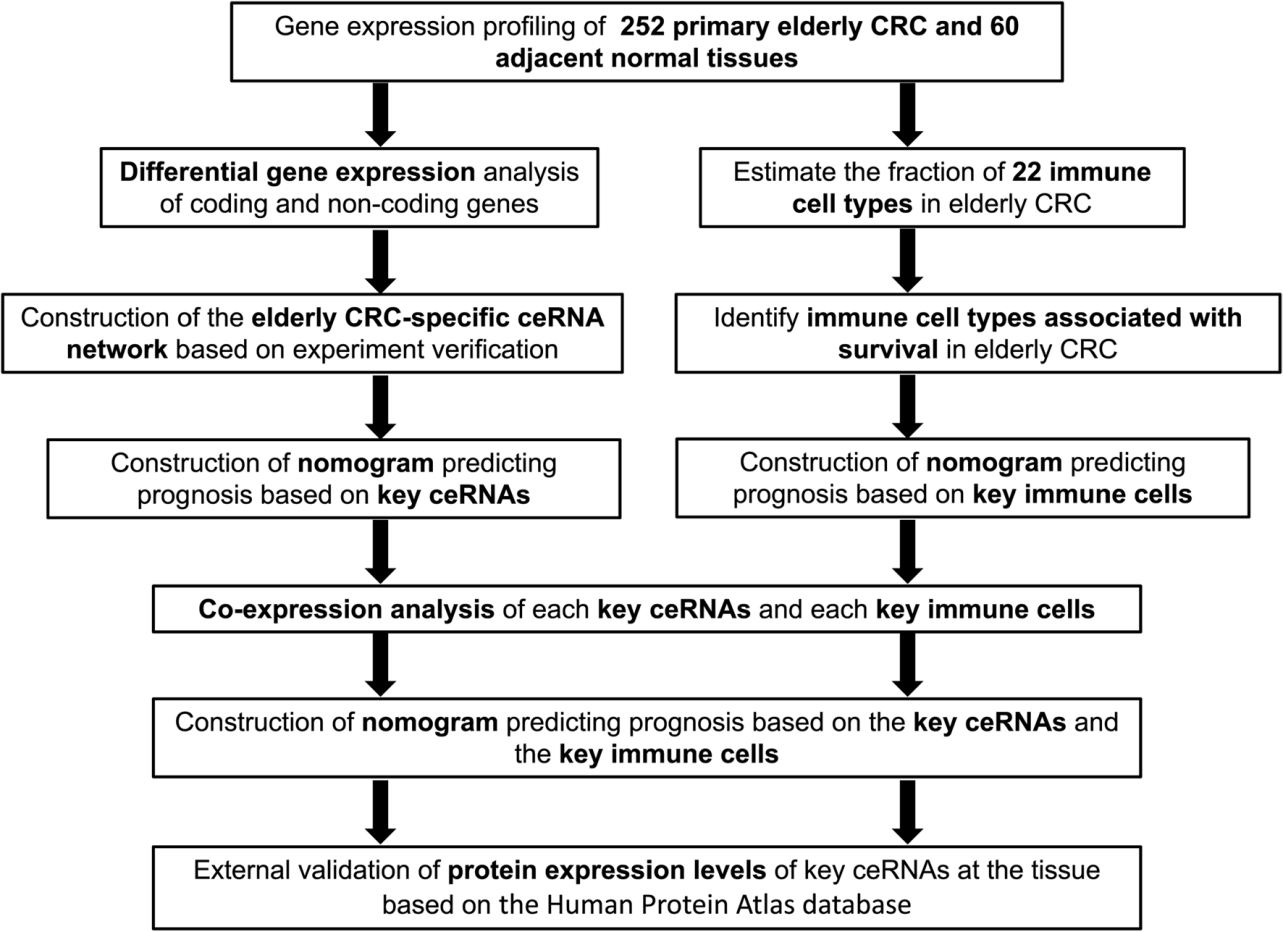
Flow chart of the experimental design and analysis process. ceRNA, competitive endogenous RNA; CRC, colorectal cancer

This study’s experimental design and analytical process are shown in Fig. 1. We included 265 elderly patients with primary CRC from the TCGA database (Table 1). By employing differential expression analysis between tumor and normal samples, we identified a total of 2991 mRNAs (Supplementary Fig. 1A, B), 223 lncRNAs (Supplementary Fig. 1C, D), and 367 miRNAs (Supplementary Fig. 1E, F) that met the criteria of *p* < 0.05 and |log2FC| > 1.

**Table 1:** Demographics and clinical characteristics of 265 elderly patients diagnosed with colorectal cancer

Characteristics	Alive (n = 197)	Dead (n = 68)	Total (n = 265)
Age, years			
Mean ± SD	74.9 ± 6.7	76.1 ± 6.4	75.2 ± 6.6
Median (IQR)	74 (69, 80)	75.5 (71, 80.3)	75 (69, 80)
Gender			
Female	89 (45.2%)	28 (41.2%)	117 (44.2%)
Male	108 (54.8%)	40 (58.8%)	148 (55.8%)
TNM stage			
I	42 (21.3%)	5 (7.4%)	47 (17.7%)
II	91 (46.2%)	21 (30.9%)	112 (42.3%)
III	48 (24.4%)	20 (29.4%)	68 (25.7%)
IV	16 (8.1%)	22 (32.4%)	38 (14.3%)
Overall survival time, days			
Mean ± SD	851.7 ± 727.5	663.9 ± 712.3	803.5 ± 726.9
Median (IQR)	701 (426, 1089)	410 (145, 1094.5)	646 (346, 1093)

*SD* standard deviation, *IQR* interquartile range

### Construction of a ceRNA network and survival analysis

**Fig. 2:**
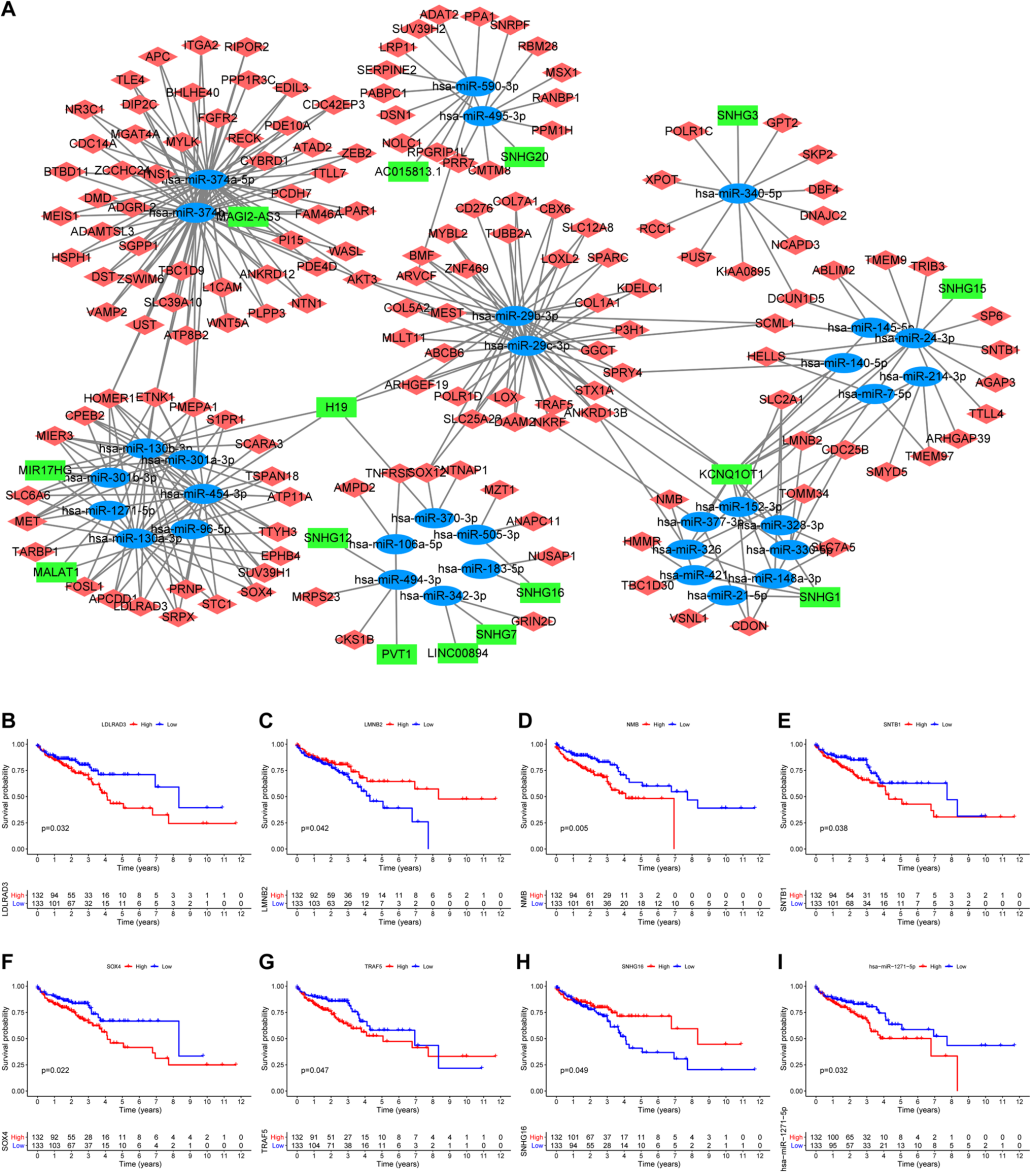
Construction of a ceRNA network and survival analysis in elderly patients with CRC. A ceRNA network consisting of 17 lncRNAs, 35 miRNAs, and 5 mRNAs (**A**). Kaplan-Meier survival curves of LDLRAD3 (**B**), LMNB2 (**C**), NMB (**D**), SNTB1 (**E**), SOX4 (**F**), TRAF5 (**G**), SNHG16 (**H**), and has-mir-1271-5p (**I**). *p* < 0.05. ceRNA, competitive endogenous RNA; CRC, colorectal cancer; lncRNA: long non-coding RNA; miRNA: microRNA; mRNA, messenger RNA

Based on the differentially expressed lncRNAs, miRNAs, and mRNAs, we constructed a ceRNA network consisting of 17 lncRNAs, 35 miRNAs, and 5 mRNAs, all of which met the criteria of the hypergeometric test and correlation analysis (*p* < 0.05) (Fig. 2A and Supplementary Table 1). Subsequently, we explored the prognostic value of the genes in the ceRNA network in elderly patients with CRC. We performed Kaplan-Meier survival analysis and found that the expression levels of *LDLRAD3* (*p* = 0.032), *LMNB2* (*p* = 0.042), *NMB* (*p* = 0.005), *SNTB1* (*p* = 0.038), *SOX4* (*p* = 0.022), *TRAF5* (*p* = 0.047), SNHG16 (*p* = 0.049) and *has-mir-1271-5p* (*p* = 0.032) could influence the prognosis in elderly patients with CRC (Fig. 2B–H).

### Development of a ceRNA signature and construction of a nomogram based on key genes in the ceRNA signature

**Fig. 3:**
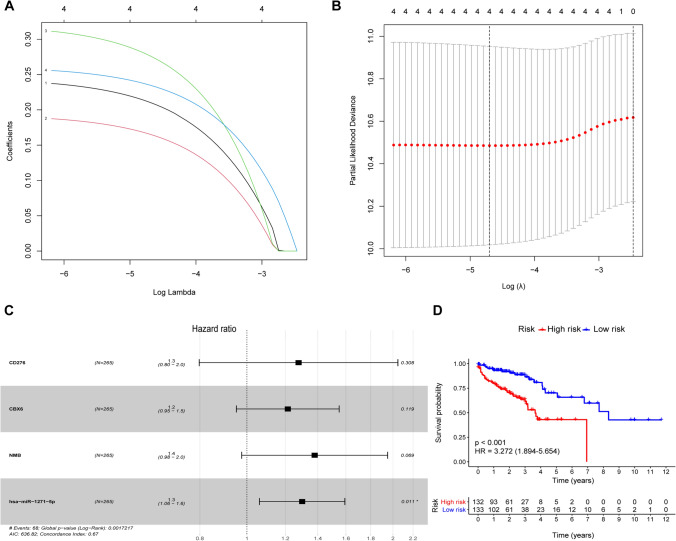
Development of a ceRNA signature for prognosis prediction. Tenfold cross-validation was performed to select the tuning parameter (lambda) in the LASSO Cox model based on the minimum criteria for OS (**A**). The LASSO coefficient profiles of survival-related ceRNAs (**B**). Forest plot of four key ceRNAs based on multivariate Cox regression analysis (**C**). Kaplan-Meier curves of OS for patients in the high- and low-risk groups (**D**). *p* < 0.05. ceRNA, competitive endogenous RNA; HR, hazard ratio; LASSO, the least absolute shrinkage and selection operator method; OS, overall survival

Using univariate Cox regression analysis, we detected four key ceRNA genes (Table 2). In addition, LASSO Cox regression indicated the lack of overfitting among these four key genes, which could be incorporated into a multivariate stepwise regression (Fig. 3A–B). We thus constructed the ceRNA prognostic signature involving four key genes (*CD276*, *CBX6*, *NMB*, and *has-miR-1271-5p*) using a multivariate stepwise regression method (Fig. 3C). Consecutively, we extracted the coefficient and expression values of the four ceRNAs to calculate the risk score for each patient using the following formula:

risk score = (0.245 × level of *CD276*) + (0.194 × level of *CBX6*) + (0.321 × level of *NMB*) + (0.262 × level of *miR-1271-5p*).

We calculated the risk scores for each patient according to the above formula, and divided them into a high- (n = 132) and low-risk (n = 133) group based on the median risk score (0.984). Using Kaplan-Meier survival curve analysis, we observed that the high-risk group had worse OS than the low-risk group (hazard ratio (HR) = 3.272, 95% confidence interval (95% CI) = 1.894–5.654, *p* < 0.001) (Fig. 3D).

To establish a quantitative method for predicting prognosis in elderly patients with CRC, we established a nomogram (ceRNA nomogram) based on the four key ceRNAs (Supplementary Fig. 2A). We noticed that the C-index of the nomogram was 0.665. We then applied both ROC and calibration curve analyses, which indicated this nomogram did not possess good accuracy (1-year AUC: 0.743, 3-year AUC: 0.764, 5-year AUC: 0.761) (Supplementary Fig. 2B) and calibration (Supplementary Fig. 2C–E).

**Table 2:** Univariate Cox regression analysis of OS based on ceRNAs

Gene	HR	95%CI	p-value
CD276	1.642	1.045–2.580	0.031*
CBX6	1.270	1.012–1.594	0.039*
NMB	1.447	1.025–2.042	0.036*
hsa-miR-1271-5p	1.344	1.094–1.650	0.005*

*OS* overall survival, *HR* hazard ratio, *CI* confidence interval

* p < 0.05. ceRNA, competitive endogenous RNA

### Composition of infiltrating immune cells between tumor and normal samples in elderly patients with CRC

**Fig. 4:**
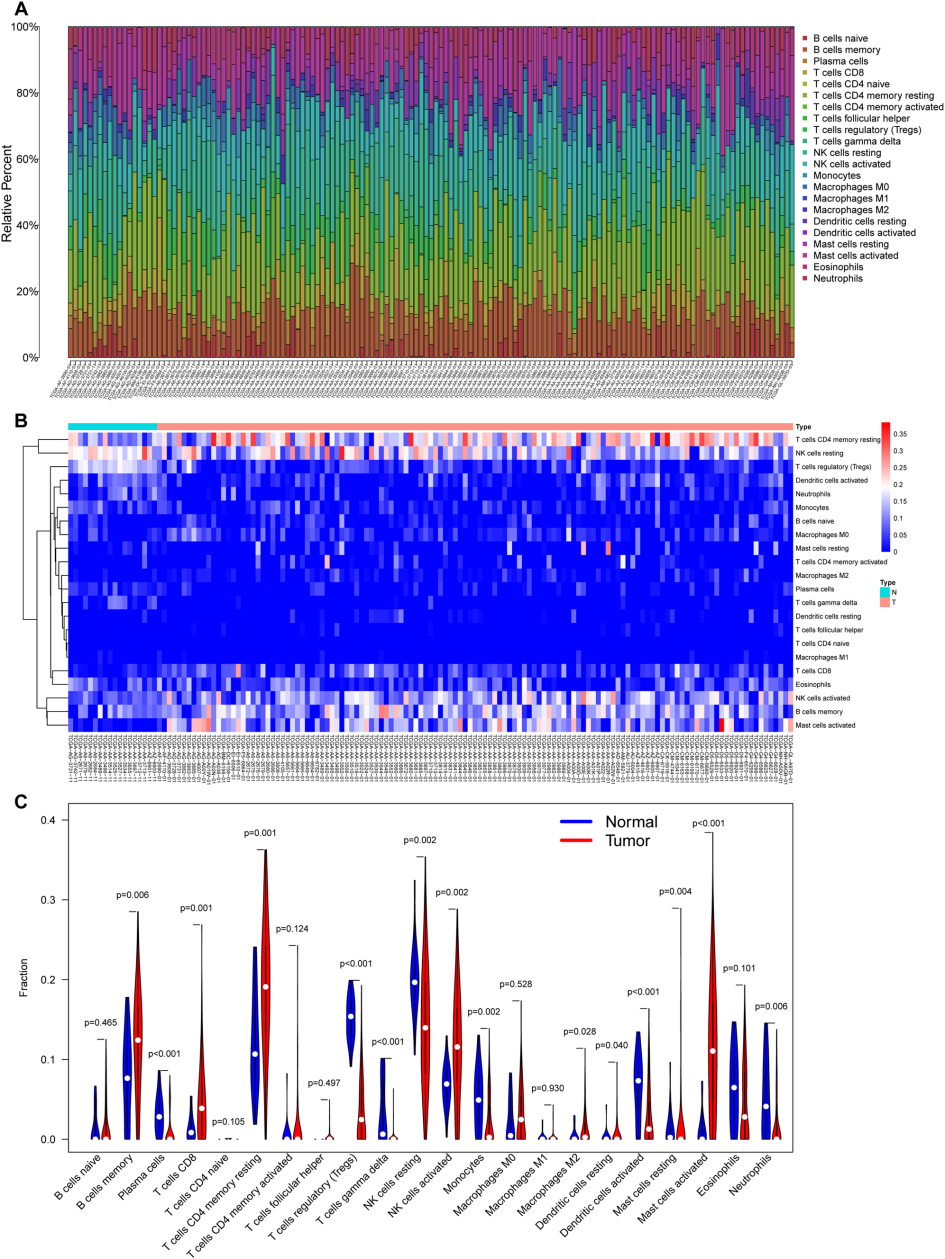
Composition of infiltrating immune cells between tumor and normal samples. Percentage stacked bar chart (**A**) and heatmap (**B**) showing the distribution of the 22 immune cells assessed by the CIBERSORT algorithm in CRC samples from elderly patients. Violin plots were used to visualize the differences in immune cell abundance between tumor and normal samples. Tumor samples are represented in red, while normal samples are represented in blue (**C**). *p* < 0.05. CIBERSORT, cell type identification by estimating relative subsets of RNA transcripts; *CRC* colorectal cancer

We performed the CIBERPORT algorithm to calculate the proportions of the 22 immune cell types in each sample of elderly patients with CRC (Fig. 4A), and visualized the level of expression of immune cells in cancer and paired normal samples using a heatmap (Fig. 4B). Wilcoxon rank-sum test revealed a significant increase in the proportion of Palma cells (*p* < 0.001), regulatory T cells (*p* < 0.001), gamma delta T cells (*p* < 0.001), resting NK cells (*p* = 0.002), monocytes (*p* = 0.002), resting dendritic cells (*p* = 0.040), activated dendritic cells (*p* < 0.001), and neutrophils (*p* = 0.006) in normal samples compared with those in tumor samples. However, we found that memory B cells (*p* = 0.006), CD8 T cells (*p* = 0.001), memory resetting CD4 T cells (*p* = 0.001), activated NK cells (*p* = 0.002), M2 macrophages (*p* = 0.028), and activated mast cells (*p* < 0.001) were significantly more abundant in tumor than in normal samples (Fig. 4C). These results collectively suggested that the altered abundance of immune cells in elderly patients with CRC might disturb the tumor microenvironment.

### Development of an immune cell signature and construction of a nomogram based on key cells in the immune cell signature

**Fig. 5:**
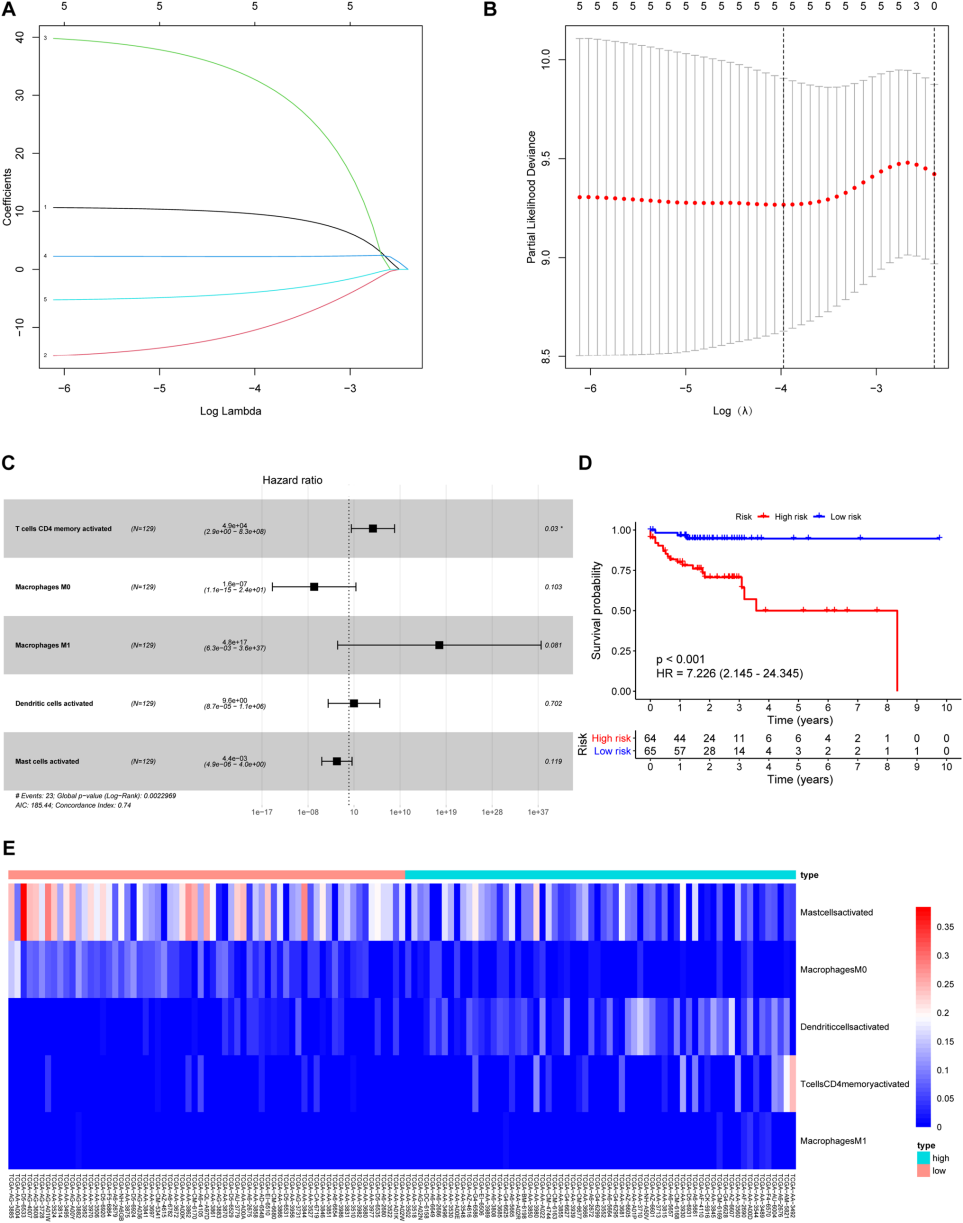
Development of an immune cell signature for prognosis prediction. Tenfold cross-validation was performed to select the tuning parameter (lambda) in the LASSO Cox model based on the minimum criteria for OS (**A**). The LASSO coefficient profiles of survival-related immune cells (**B**). Forest plot of five key immune cells based on multivariate Cox regression analysis (**C**). Kaplan-Meier curves of OS for patients in high- and low-risk groups (**D**). Heatmap (**E**) showing the distribution of the five key immune cells in the high- and low-risk groups. *p* < 0.05. ceRNA, competitive endogenous RNA; HR, hazard ratio; LASSO, the least absolute shrinkage and selection operator method; OS, overall survival

Using univariate Cox regression analysis of the 22 immune cells, we identified five immune cells that influenced prognosis. We did not detect any overfitting among the five immune cells, as indicated by the LASSO Cox regression analysis (Fig. 5A–B). We constructed the immune cell signature involving a total of five key immune cells (memory activated CD4 T cells, activated dendritic cells, M0 macrophages, M1 macrophages, and activated mast cells) using a multivariate stepwise regression method (Fig. 5C). Subsequently, patients were divided into high- and low-risk groups based on the median risk score obtained from the immune cell signature. Using Kaplan-Meier survival analysis, we noticed that elderly patients with CRC in the high-risk group had worse OS compared with those in the low-risk group (HR = 7.226, 95% CI = 2.145–24.345, *p* < 0.001) (Fig. 5D). The heatmap showed that the levels of activated mast cells and M0 macrophages were higher, whereas those of activated dendritic cells, activated memory CD4 T cells, and M1 macrophages were lower in the high-risk group than in low-risk group (Fig. 5E).

We accordingly constructed a nomogram (immune cell nomogram) based on these five key immune cells in the immune cell signature to predict prognosis in elderly patients with CRC (Supplementary Fig. 3A). We detected that the C-index of the nomogram was 0.736. We then applied both ROC and calibration curve analyses, which manifested an acceptable accuracy (1-year AUC: 0.671, 3-year AUC: 0.688, 5-year AUC: 0.668) (Supplementary Fig. 3B) and calibration of the nomogram (Supplementary Fig. 3C–E).

### Correlation analysis of key ceRNAs with immune cells

**Fig. 6:**
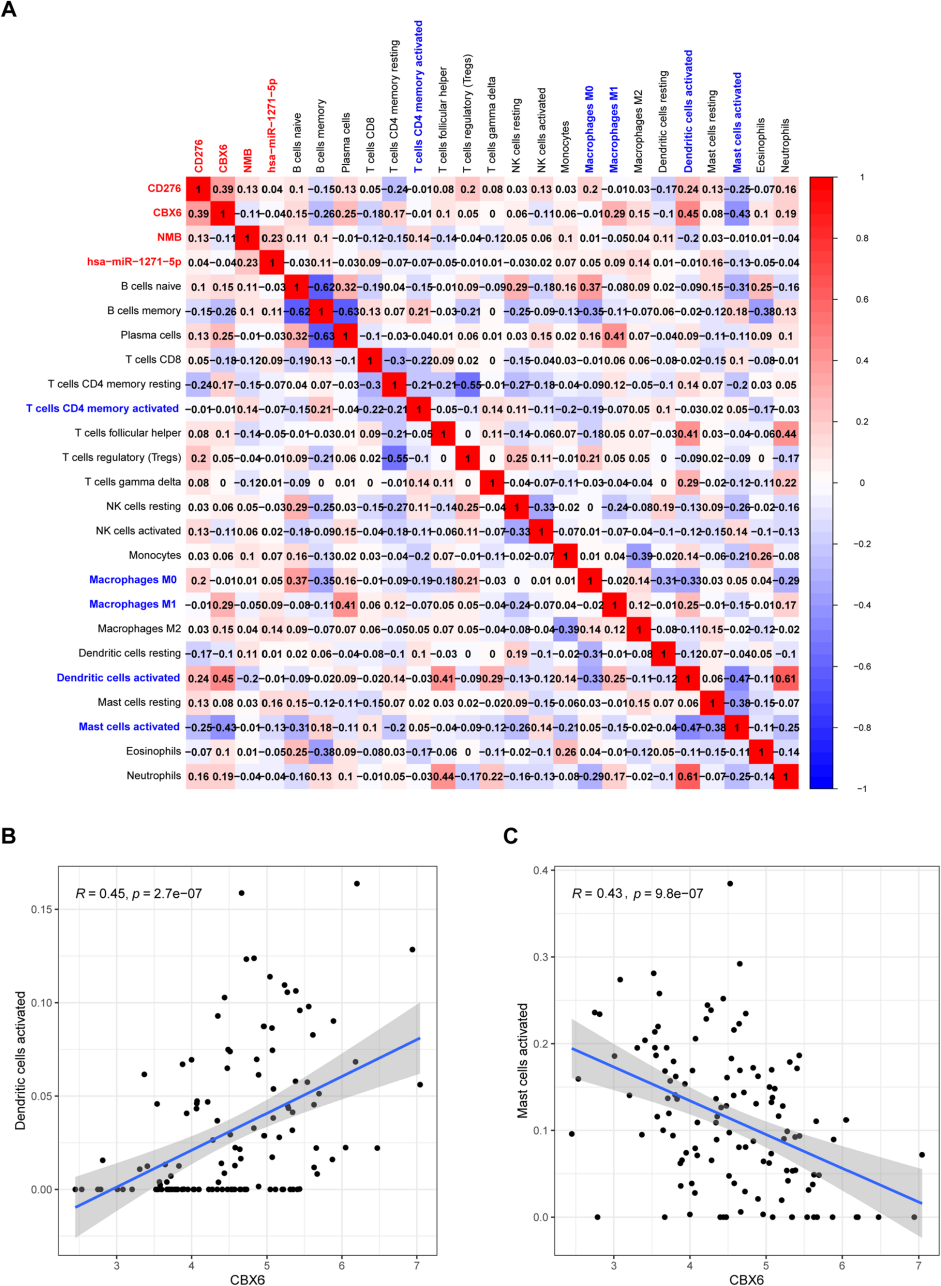
Correlation analysis of four key ceRNAs with immune cells. Correlation matrix of the four key ceRNAs with 22 tumor-infiltrating immune cells. Red represents positive correlation, whereas blue represents negative correlation (**A**). *CBX6* was positively correlated with activated dendritic cells (**B**), whereas it was negatively correlated with activated mast cells (**C**). *p* < 0.05. ceRNA, competitive endogenous RNA.

To further explore whether ceRNAs and immune cells were related, we visualized the co-expression patterns of four key ceRNAs and 22 immune cells using a correlation heatmap (Fig. 6A). Pearson correlation analysis revealed that *CBX6* was positively correlated with activated dendritic cells (*R* = 0.45, *p* < 0.01), whereas it was negatively correlated with activated mast cells (*R* =− 0.43, *p* < 0.01) (Fig. 6B–C). Overall, we found that these markers were strongly correlated with the overall level of the tumor immune microenvironment in elderly patients with CRC.

### Survival analysis and construction of a nomogram based on ceRNA-immune cell signature

**Fig. 7:**
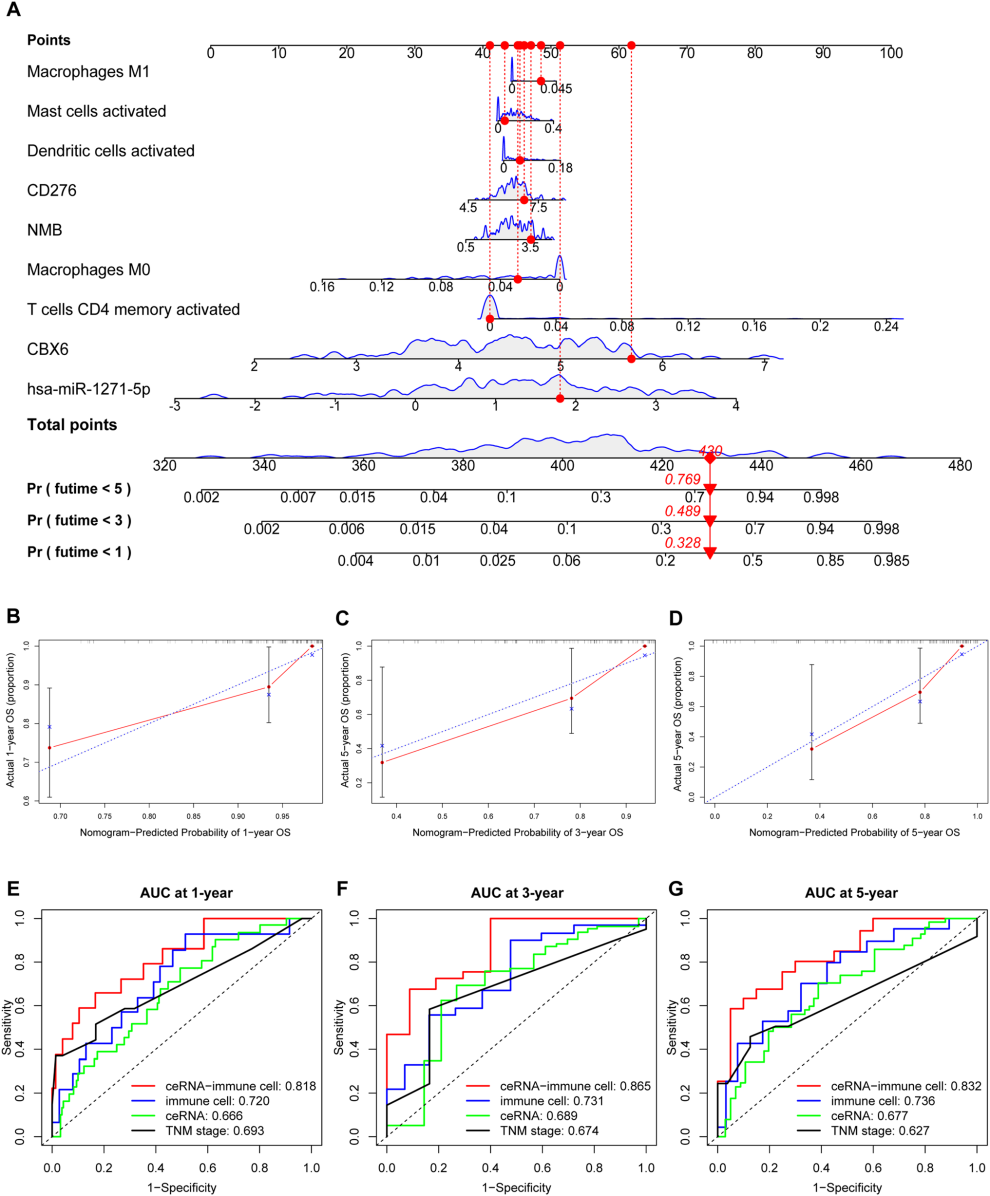
Construction of a nomogram based on the ceRNA-immune cell signature. Nomogram constructed by combining the four key ceRNAs and five key immune cells (**A**). The red dot represents an example of a single elderly patient. Calibration plots for predicting the 1- (**B**), 3- (**C**), and 5-years (**D**) OS. Comparison of the AUC values of the three nomograms and TNM stages based on time-dependent ROC curve analysis at 1- (**E**), 3- (**F**), and 5-years (**G**) OS. AUC, area under the curve; ceRNA, competitive endogenous RNA; OS, overall survival; ROC, receiver operating characteristic

We found that the ceRNA and immune cell signatures could independently predict prognosis in elderly patients with CRC. Therefore, assuming that combining these two signatures could result in better prediction accuracy, we integrated the four key ceRNAs and five key immune cells to develop a ceRNA-immune cell signature. According to the median risk score of the signature, patients were divided into high- and low-risk groups. Through Kaplan-Meier survival analysis, it was observed that elderly patients with CRC in the high-risk group had a poorer OS compared to those in the low-risk group (HR = 7.650, 95% CI = 2.271–25.770, *p* < 0.001) (Supplementary Fig. 4). Then, we constructed a combined nomogram (ceRNA-immune cell nomogram), including the four key ceRNAs and five key immune cells (Fig. 7A). We accordingly observed that the calibration curves for 1-, 3-, and 5-years OS were close to the standard curve, indicating good model performance (Fig. 7B–D). The results of C-index (C-index = 0.807 vs. 0.665 vs. 0.736) and AUCs (1-year AUC: 0.818 vs. 0.720 vs. 0.666, 3-year AUC: 0.865 vs. 0.731 vs. 0.689, 5-year AUC: 0.832 vs. 0.736 vs. 0.627) showed that the ceRNA-immune cell nomogram had better accuracy and consistency than either the immune cell nomogram or the ceRNA nomogram alone (Fig. 7E–G). Although the TNM stage is the most widely used clinical prognosis-related predictor, we noticed that the AUCs of the ceRNA-immune cell nomogram were significantly greater than those of the TNM stage at 1 (0.818 vs. 0.693), 3 (0.865 vs. 0.674), and 5 (0.832 vs. 0.627) years, indicating that our ceRNA-immune cell nomogram had better prognostic accuracy (Fig. 7E–G).

### External validation of the protein expression level corresponding to mRNAs

**Fig. 8:**
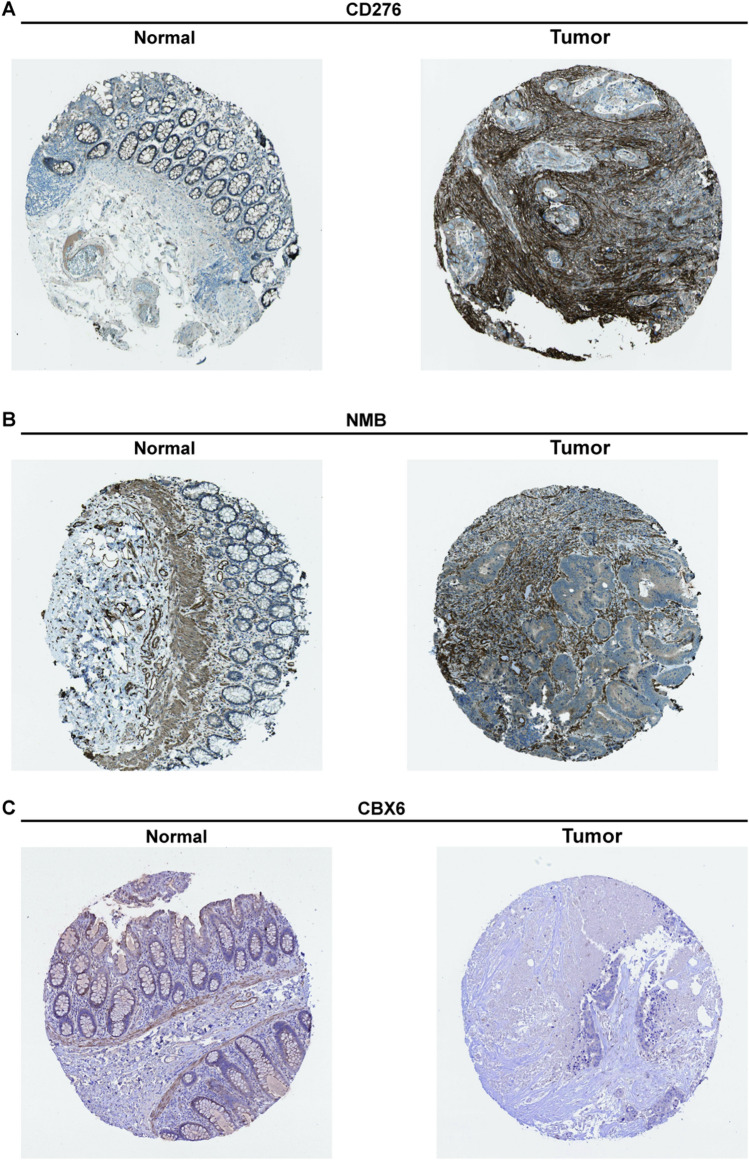
External validation of protein expression levels corresponding to mRNAs in the Human Protein Atlas database. Comparison of the level of protein expression of CD276 (**A**), NMB (**B**) and CBX6 (**C**) in normal and tumor tissues.

The expression level of mRNAs (*CD276*, *NMB*, and *CBX6*) in the ceRNA signature was confirmed by the sequencing data in TCGA. As already known, mRNA performs its function through protein; we thus used the Human Protein Atlas database for external validation of the expression of proteins corresponding to these three mRNAs in normal and tumor tissues of elderly patients with CRC. Our results revealed higher expression of the CD276 and NMB proteins in tumor than in normal tissues (Fig. 8A, B), whereas CBX6 was only highly expressed in normal intestinal mucosal tissues (Fig. 8C). The results are consistent with our analysis at the mRNA level.

## Discussion

New cases of CRC mainly occur in elderly patients [24, 25]. Various molecular and genetic biomarkers, including protein-coding genes, non-coding genes, and immune cells, are utilized for predicting the prognosis and identifying potential treatment targets in CRC [21, 26, 27]. Among these biomarkers, ceRNAs and tumor-infiltrating immune cells have recently emerged as potential candidates. However, little research has focused on their action in elderly patients with CRC.

Therefore, we aimed to identify and analyze a novel ceRNA network and related immune cells for prognosis prediction and clinical treatment guidance in elderly patients with CRC. We found differently expressed ceRNAs and immune cells between normal and tumor samples in elderly patients with CRC. Subsequently, we established three prognosis predictive nomograms based on four key ceRNAs (ceRNA nomogram), five key immune cells (immune cell nomogram) or their combination (ceRNA-immune cell nomogram). Among them, the ceRNA-immune cell nomogram had the best accuracy and consistency compared with others, which might be used to predict the 1-, 3-, and 5-year OS of patients and guide their clinical treatment.

In recent years, increasing studies have revealed the involvement of ceRNAs in the tumorigenesis and their potential as promising predictive prognosis biomarkers [28]. The present study used bioinformatic analysis to identify the ceRNA networks. Using univariate, LASSO, and multivariate Cox regression analyses, we developed a ceRNA prognostic signature involving four key genes (*CD276, CBX6, NMB*, and *has-miR-1271-5p*). As a key member of the B7 superfamily, *CD276* is highly expressed in hepatocellular carcinoma, lung cancer, and adrenocortical carcinoma [29–31]. Takashima et al. [32] also found that *CD276* was effective for the prognosis of glioblastoma multiforme. The expression of the *CBX* family proteins has been reported in a variety of malignancies [33]. Furthermore, *CBX6* is known to regulate gene expression, cell replication, and differentiation, and has been demonstrated to play a significant role in hepatocellular carcinoma [34, 35]. *NMB* is a single transmembrane protein expressed in differentiated immune cells. Metz et al. [36] demonstrated that NMB has properties of enhancing tumorigenesis and might thus be the core component in the development of malignant tumors. In addition, *has-miR-1271-5p* was found to play a critical role in cancer development [37, 38]. These findings were consistent with those of our study.

Previous studies have revealed that tumor-infiltrating immune cells play a vital role in determining the prognosis of solid tumors [39, 40]. In our study, using the method as that used for constructing the ceRNA signature, we constructed an immune cell prognostic signature involving five immune cells (activated memory CD4 T cells, activated dendritic cells, M0 macrophages, M1 macrophages, and activated mast cells). Notably, the activated memory CD4 T cells were identified as independent prognostic factors. Novy et al. [41] found that activated memory CD4 T cells influence tumor growth by affecting the function of CD8 T cells. Several studies have also found that activated memory CD4 T cells are associated with prognoses in many cancers, such as breast cancer, cervical cancer, non-small cell lung cancer, and pancreatic adenocarcinomas [42–45].

Our co-expression analysis revealed that the expression of *CBX6* was positively associated with activated dendritic cells (*R* = 0.45, *p* < 0.01), but negatively associated with activated mast cells (*R* =-0.43, *p* < 0.01). Previous studies also found that the expression of *CBX6* was significantly correlated with the infiltration of dendritic cells in carcinoma [46, 47]. Concomitantly, a series of studies have reported that the number of mast cells in the tumor is related to prognosis [48–50]. In our study, we showed that the expression of *CBX6* was closely related to the infiltration of mast cells, suggesting that CBX6 might reflect not only disease prognosis but also immune status. Thus, we deduced that the two pairs and their relevant mechanisms would play essential roles in the prognosis of elderly patients with CRC. We presumed that *CBX6* might regulate the populations of activated dendritic cells and activated mast cells in the tumor immune microenvironment, in turn affecting the development of tumors in elderly patients with CRC.

As mentioned above, both ceRNAs and immune cells play an important role in tumor development and prognosis. We here found that the ceRNA and immune cell nomograms could independently predict prognosis in elderly patients with CRC. Therefore, assuming that their combination could result in better prediction accuracy, we developed a ceRNA-immune cell nomogram. We found this had better accuracy and discrimination than the others, as evaluated based on the C-index, ROC analysis, and calibration curves. The ceRNA-immune cell nomogram would be valuable to elderly patients with CRC and colorectal surgeons because it could facilitate the accurate evaluation of the 1-, 3-, and 5-year OS after surgical treatment and provide more comprehensive information for the improved guidance of personalized clinical treatment.

Inevitably, our study has some limitations. As this analysis was conducted retrospectively using public databases and it is challenging to avoid selection bias in such settings, further external cohorts are required to validate these results. Moreover, in vivo and in vitro experiments are needed to further study the mechanism of prognostic ceRNAs and immune cells in elderly patients with CRC.

## Conclusions

In conclusion, our study constructed three nomograms based on the ceRNA network and tumor-infiltrating immune cells to predict prognosis in elderly patients with CRC. Among them, the ceRNA-immune cell nomogram had the best prediction accuracy. Moreover, our study inferred that the mechanism underlying the regulation of activated dendritic cells and activated mast cells by *CBX6* might playa crucial role in tumor development and prognosis in elderly patients with CRC.

## Electronic supplementary material

Supplementary Material 1

## Data Availability

The gene expression profiling data were downloaded from The Cancer Genome Atlas (TCGA) (https://tcga-data.nci.nih.gov/tcga/). The datasets used and/or analyzed in the current study are available from the corresponding author upon reasonable request.

## Abbreviations

AUC: Area under the curve
ceRNA: Competitive endogenous RNA
C-index: Concordance index
CRC: Colorectal cancer
FC: Fold change
FDR: False discovery rate
HR: Hazard ratio
IQR: Interquartile range
LASSO: Least absolute shrinkage and selection operator method
lncRNA: Long non-coding RNA
miRNA: MicroRNA
mRNA: Messenger RNA
OS: Overall survival
ROC: Receiver operating characteristic
SD: Standard deviation
TCGA: The Cancer Genome Atlas

## Code availability

The code used in the current study are available from the corresponding author upon reasonable request.
