# Supplementary material for: Construction of a novel nomogram based on competing endogenous RNAs and tumor-infiltrating immune cells for prognosis prediction in elderly patients with colorectal cancer
Source: Discov Oncol. 2023 Jul 10;14:125. doi: 10.1007/s12672-023-00742-y (PMC10333159; doi:10.1007/s12672-023-00742-y)
Supplement: Supplementary file 1 — Supplementary Material 1 [file 12672_2023_742_MOESM1_ESM.docx]

**Supplementary Material**


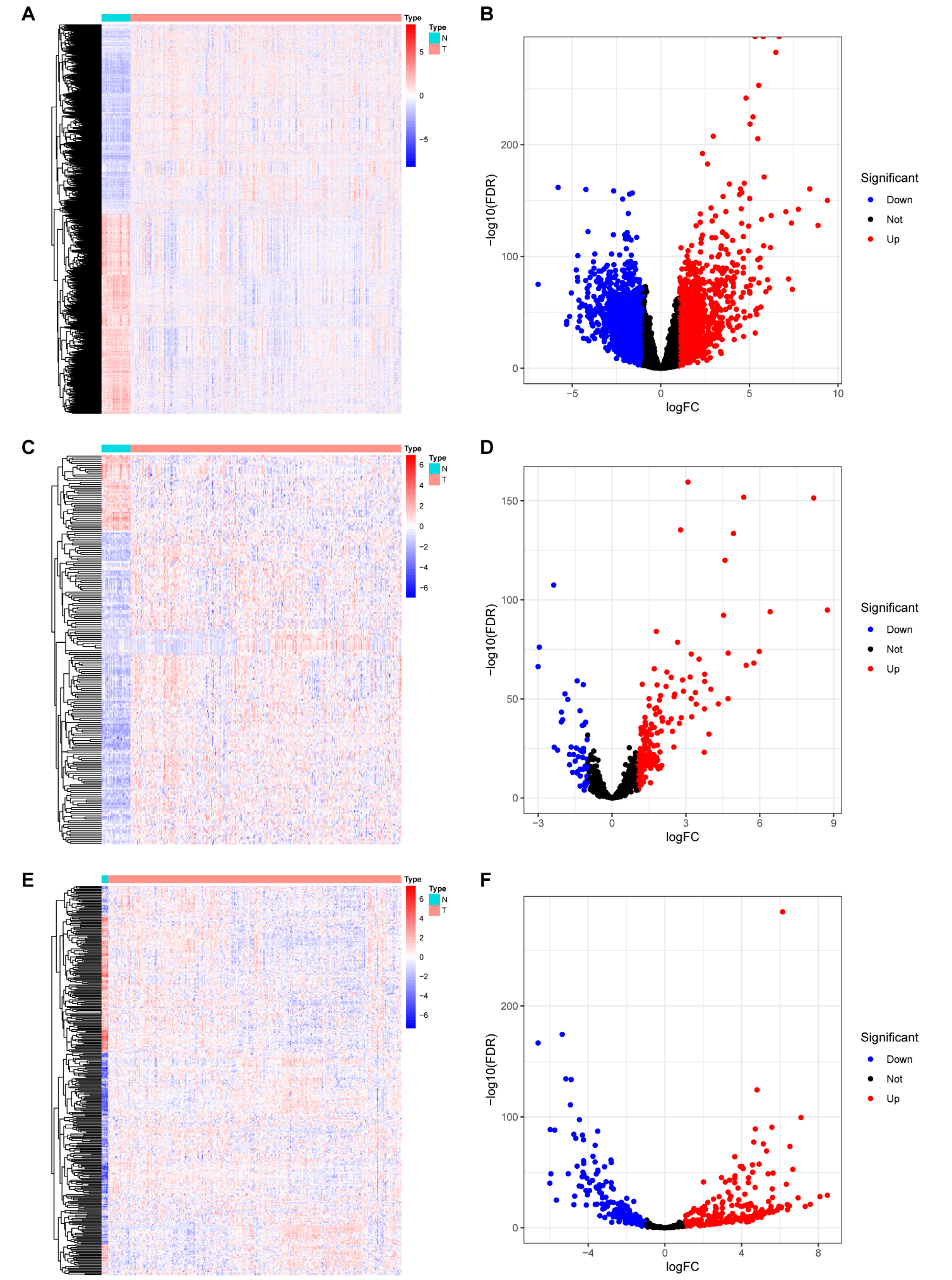


**Supplementary Figure 1**. **Identification of differentially expressed mRNAs, lncRNAs, and miRNAs between tumor and normal samples.** Heatmap and volcano plot of 2991 differentially expressed mRNAs (**A, B**), 223 differentially expressed lncRNAs (**C, D**), and 367 differentially expressed miRNAs (**E, F**). The threshold was set at FDR < 0.05 and |log2FC| > 1. FC: fold change; FDR: false discovery rate; lncRNA: long non-coding RNA; miRNA: microRNA; mRNA, messenger RNA.


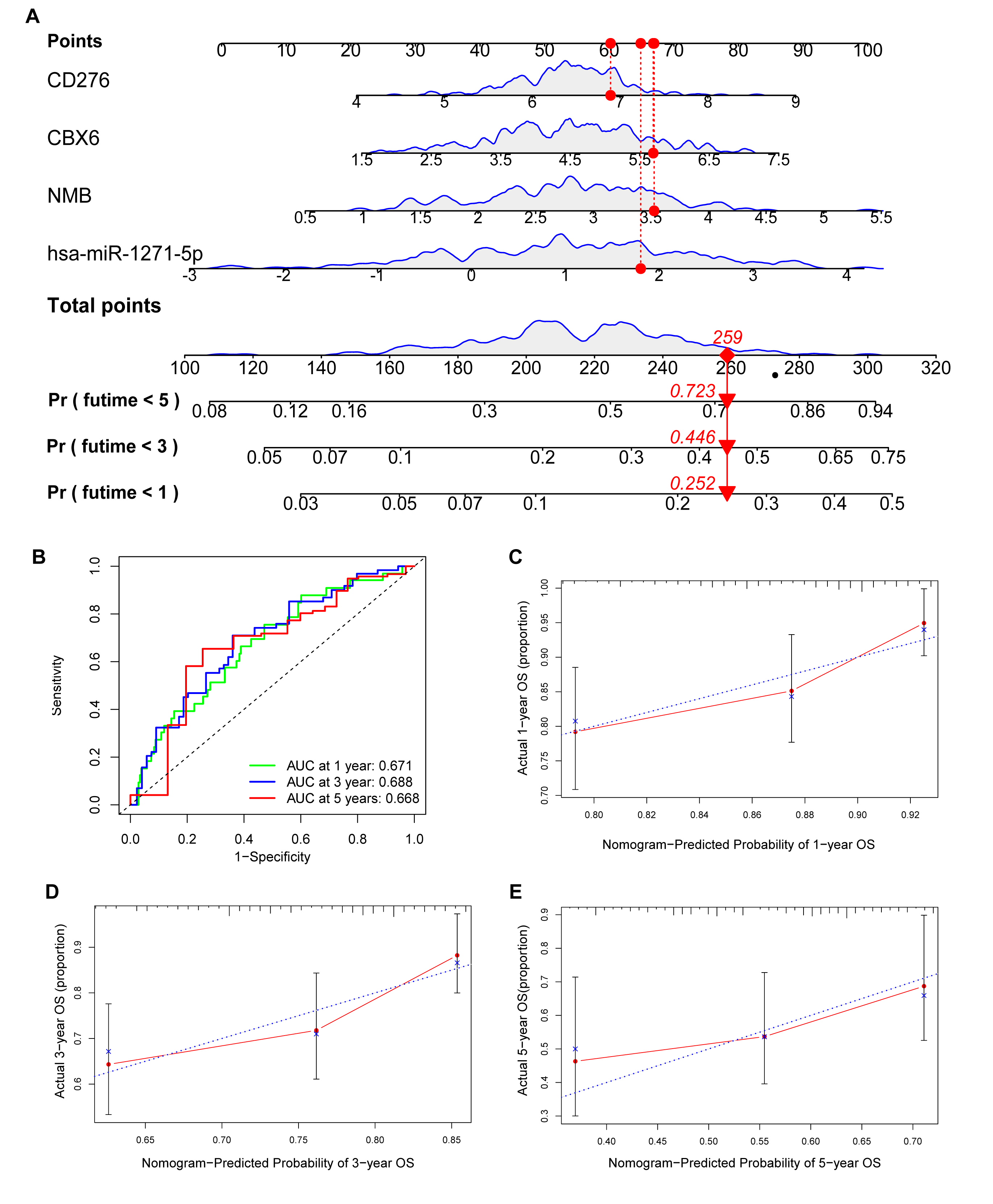


**Supplementary Figure 2. Construction of a nomogram based on key genes in the ceRNA signature.** Nomogram constructed by combining four key genes in the ceRNA signature (**A**). The red dot represents an example of a single elderly patient. Time-dependent ROC curve analysis at 1-, 3-, and 5-years OS (**B**). Calibration plots for predicting the 1- (**C**), 3- (**D**), and 5-years (**E**) OS. AUC, area under the curve; ceRNA, competitive endogenous RNA; OS, overall survival; ROC, receiver operating characteristic.

**
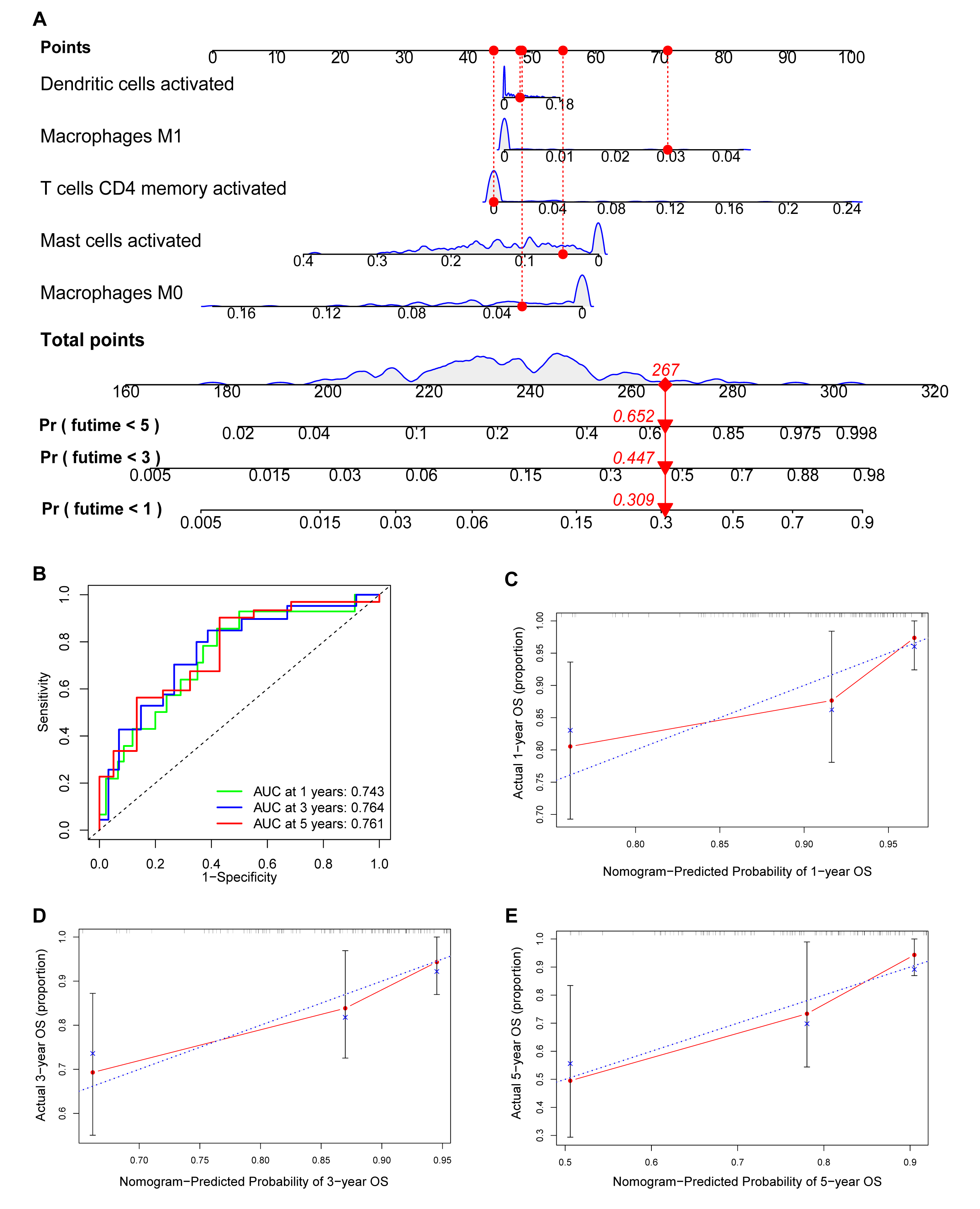
**

**Supplementary Figure 3. Construction of a nomogram based on key cells in the immune cell signature.** Nomogram constructed by combining the five key immune cells in the immune cell signature (**A**). The red dot represents an example of a single elderly patient. Time-dependent ROC curve analysis at 1-, 3-, and 5-years OS (**B**). Calibration plots for predicting the 1- (**C**), 3- (**D**), and 5-years (**E**) OS. AUC, area under the curve; OS, overall survival; ROC, receiver operating characteristic.


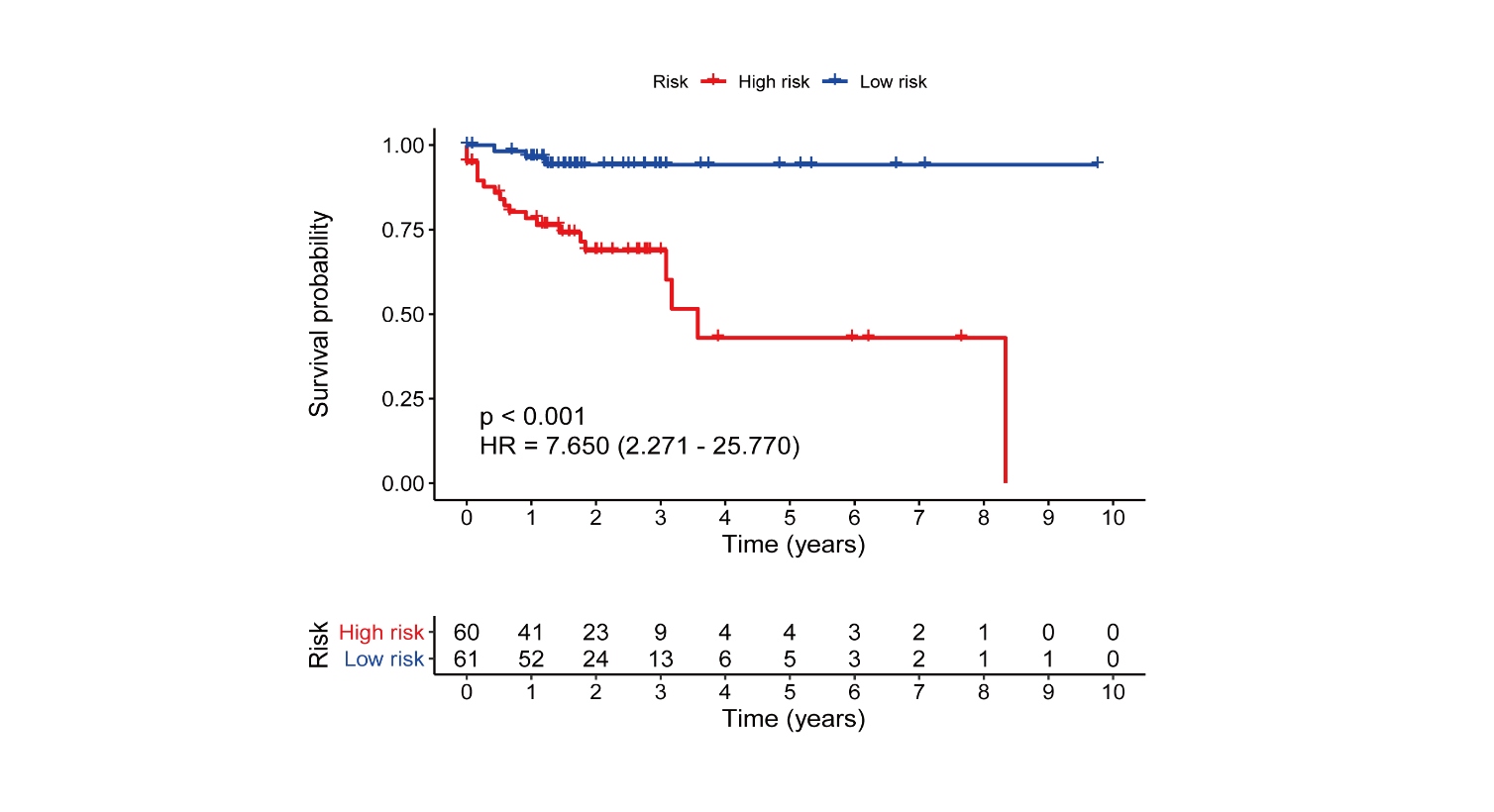


**Supplementary Figure 4**. **Survival analysis of ceRNA-immune cell signature.** *p* < 0.05. HR, hazard ratio.

**Supplementary Table 1: Hypergeometric testing and correlation analysis results of ceRNAs network.**

| **LncRNAs** | **Protein-coding RNAs** | **MiRNAs** | **Hypergeometric test P** | **Correlation P** |
| --- | --- | --- | --- | --- |
| PVT1 | AMPD2 | hsa-miR-106a-5p | 0.027 | 0.000 |
| PVT1 | TNFRSF10B | hsa-miR-106a-5p | 0.005 | 0.046 |
| SNHG15 | TRIB3 | hsa-miR-24-3p | 0.036 | 0.000 |
| SNHG15 | TOMM34 | hsa-miR-24-3p | 0.043 | 0.000 |
| SNHG15 | SNTB1 | hsa-miR-24-3p | 0.043 | 0.000 |
| SNHG15 | DCUN1D5 | hsa-miR-24-3p | 0.025 | 0.000 |
| SNHG15 | TTLL4 | hsa-miR-24-3p | 0.047 | 0.000 |
| SNHG15 | AGAP3 | hsa-miR-24-3p | 0.036 | 0.000 |
| SNHG15 | HELLS | hsa-miR-24-3p | 0.014 | 0.000 |
| SNHG15 | LMNB2 | hsa-miR-24-3p | 0.014 | 0.000 |
| SNHG15 | SCML1 | hsa-miR-24-3p | 0.036 | 0.000 |
| SNHG15 | SP6 | hsa-miR-24-3p | 0.011 | 0.001 |
| SNHG15 | ARHGAP39 | hsa-miR-24-3p | 0.004 | 0.000 |
| SNHG15 | TMEM9 | hsa-miR-24-3p | 0.022 | 0.000 |
| SNHG15 | ABLIM2 | hsa-miR-24-3p | 0.007 | 0.003 |
| SNHG1 | TOMM34 | hsa-miR-326,hsa-miR-330-5p,hsa-miR-421 | 0.001 | 0.000 |
| SNHG1 | ANKRD13B | hsa-miR-326,hsa-miR-330-5p | 0.041 | 0.000 |
| SNHG1 | VSNL1 | hsa-miR-21-5p | 0.043 | 0.000 |
| SNHG1 | NMB | hsa-miR-421 | 0.043 | 0.000 |
| SNHG1 | TBC1D30 | hsa-miR-421 | 0.022 | 0.000 |
| SNHG1 | LMNB2 | hsa-miR-326,hsa-miR-330-5p | 0.002 | 0.003 |
| SNHG1 | HMMR | hsa-miR-421 | 0.043 | 0.000 |
| SNHG16 | NUSAP1 | hsa-miR-183-5p | 0.014 | 0.000 |
| SNHG16 | MZT1 | hsa-miR-505-3p | 0.014 | 0.000 |
| SNHG16 | ANAPC11 | hsa-miR-505-3p | 0.014 | 0.000 |
| MIR17HG | SLC6A6 | hsa-miR-130a-3p,hsa-miR-454-3p,hsa-miR-301a-3p,hsa-miR-301b-3p,hsa-miR-130b-3p | 0.001 | 0.000 |
| MIR17HG | MIER3 | hsa-miR-130a-3p,hsa-miR-454-3p,hsa-miR-301a-3p,hsa-miR-301b-3p,hsa-miR-130b-3p | 0.000 | 0.000 |
| MIR17HG | MET | hsa-miR-130a-3p,hsa-miR-454-3p,hsa-miR-301a-3p,hsa-miR-301b-3p,hsa-miR-130b-3p | 0.000 | 0.000 |
| MIR17HG | HOMER1 | hsa-miR-130a-3p,hsa-miR-454-3p,hsa-miR-301a-3p,hsa-miR-301b-3p,hsa-miR-130b-3p | 0.000 | 0.000 |
| MIR17HG | CPEB2 | hsa-miR-130a-3p,hsa-miR-454-3p,hsa-miR-301a-3p,hsa-miR-301b-3p,hsa-miR-130b-3p | 0.001 | 0.000 |
| MIR17HG | ETNK1 | hsa-miR-130a-3p,hsa-miR-454-3p,hsa-miR-301a-3p,hsa-miR-301b-3p,hsa-miR-130b-3p | 0.005 | 0.003 |
| SNHG12 | MRPS23 | hsa-miR-494-3p | 0.018 | 0.000 |
| SNHG12 | CKS1B | hsa-miR-494-3p | 0.007 | 0.000 |
| SNHG20 | PPM1H | hsa-miR-495-3p | 0.047 | 0.000 |
| SNHG20 | PRR7 | hsa-miR-495-3p | 0.011 | 0.002 |
| SNHG20 | RBM28 | hsa-miR-495-3p | 0.004 | 0.000 |
| SNHG20 | MSX1 | hsa-miR-495-3p | 0.018 | 0.000 |
| SNHG20 | CMTM8 | hsa-miR-495-3p | 0.011 | 0.000 |
| SNHG20 | ADAT2 | hsa-miR-495-3p | 0.022 | 0.000 |
| SNHG20 | RANBP1 | hsa-miR-495-3p | 0.011 | 0.008 |
| SNHG20 | LRP11 | hsa-miR-495-3p | 0.036 | 0.000 |
| SNHG20 | SNRPF | hsa-miR-495-3p | 0.007 | 0.001 |
| SNHG3 | PUS7 | hsa-miR-340-5p | 0.029 | 0.000 |
| SNHG3 | POLR1C | hsa-miR-340-5p | 0.018 | 0.000 |
| SNHG3 | DCUN1D5 | hsa-miR-340-5p | 0.025 | 0.000 |
| SNHG3 | XPOT | hsa-miR-340-5p | 0.025 | 0.000 |
| SNHG3 | GPT2 | hsa-miR-340-5p | 0.036 | 0.000 |
| SNHG3 | NCAPD3 | hsa-miR-340-5p | 0.022 | 0.000 |
| SNHG3 | RCC1 | hsa-miR-340-5p | 0.011 | 0.000 |
| SNHG3 | DNAJC2 | hsa-miR-340-5p | 0.011 | 0.000 |
| SNHG3 | DBF4 | hsa-miR-340-5p | 0.022 | 0.000 |
| SNHG3 | SKP2 | hsa-miR-340-5p | 0.004 | 0.000 |
| SNHG3 | KIAA0895 | hsa-miR-340-5p | 0.032 | 0.000 |
| H19 | ANKRD13B | hsa-miR-29c-3p,hsa-miR-29b-3p | 0.003 | 0.000 |
| H19 | ATP11A | hsa-miR-130a-3p,hsa-miR-454-3p,hsa-miR-130b-3p | 0.002 | 0.000 |
| H19 | SOX4 | hsa-miR-130a-3p,hsa-miR-454-3p,hsa-miR-130b-3p | 0.037 | 0.022 |
| H19 | COL7A1 | hsa-miR-29c-3p,hsa-miR-29b-3p | 0.001 | 0.000 |
| H19 | TTYH3 | hsa-miR-130a-3p,hsa-miR-454-3p,hsa-miR-130b-3p | 0.001 | 0.013 |
| H19 | SRPX | hsa-miR-130a-3p,hsa-miR-454-3p,hsa-miR-130b-3p | 0.005 | 0.017 |
| H19 | EPHB4 | hsa-miR-130a-3p,hsa-miR-454-3p,hsa-miR-130b-3p | 0.001 | 0.000 |
| H19 | LDLRAD3 | hsa-miR-130a-3p,hsa-miR-454-3p,hsa-miR-130b-3p | 0.017 | 0.000 |
| H19 | DAAM2 | hsa-miR-29c-3p,hsa-miR-29b-3p | 0.001 | 0.011 |
| H19 | COL1A1 | hsa-miR-29c-3p,hsa-miR-29b-3p | 0.004 | 0.000 |
| H19 | STX1A | hsa-miR-29c-3p,hsa-miR-29b-3p | 0.005 | 0.000 |
| H19 | P3H1 | hsa-miR-29c-3p,hsa-miR-29b-3p | 0.000 | 0.000 |
| H19 | SUV39H1 | hsa-miR-130a-3p,hsa-miR-454-3p,hsa-miR-130b-3p | 0.000 | 0.005 |
| H19 | SPRY4 | hsa-miR-29c-3p,hsa-miR-29b-3p | 0.021 | 0.014 |
| H19 | STC1 | hsa-miR-130a-3p,hsa-miR-454-3p,hsa-miR-130b-3p | 0.004 | 0.000 |
| H19 | LOXL2 | hsa-miR-29c-3p,hsa-miR-29b-3p | 0.002 | 0.000 |
| H19 | CD276 | hsa-miR-29c-3p,hsa-miR-29b-3p | 0.000 | 0.000 |
| H19 | PMEPA1 | hsa-miR-130a-3p,hsa-miR-454-3p,hsa-miR-130b-3p | 0.001 | 0.041 |
| H19 | SLC25A22 | hsa-miR-29c-3p,hsa-miR-29b-3p | 0.014 | 0.000 |
| H19 | FOSL1 | hsa-miR-130a-3p,hsa-miR-454-3p,hsa-miR-130b-3p | 0.006 | 0.004 |
| H19 | ZNF469 | hsa-miR-29c-3p,hsa-miR-29b-3p | 0.021 | 0.000 |
| H19 | APCDD1 | hsa-miR-130a-3p,hsa-miR-454-3p,hsa-miR-130b-3p | 0.025 | 0.000 |
| H19 | KDELC1 | hsa-miR-29c-3p,hsa-miR-29b-3p | 0.001 | 0.025 |
| H19 | BMF | hsa-miR-29c-3p,hsa-miR-29b-3p | 0.011 | 0.002 |
| H19 | SOX12 | hsa-miR-29c-3p,hsa-miR-29b-3p,hsa-miR-370-3p | 0.001 | 0.001 |
| H19 | COL5A2 | hsa-miR-29c-3p,hsa-miR-29b-3p | 0.000 | 0.001 |
| H19 | ARVCF | hsa-miR-29c-3p,hsa-miR-29b-3p | 0.000 | 0.002 |
| H19 | SCARA3 | hsa-miR-130a-3p,hsa-miR-130b-3p | 0.005 | 0.001 |
| H19 | PRNP | hsa-miR-130a-3p,hsa-miR-454-3p,hsa-miR-130b-3p | 0.014 | 0.049 |
| H19 | MLLT11 | hsa-miR-29c-3p,hsa-miR-29b-3p | 0.001 | 0.000 |
| H19 | TUBB2A | hsa-miR-29c-3p,hsa-miR-29b-3p | 0.000 | 0.047 |
| H19 | SPARC | hsa-miR-29c-3p,hsa-miR-29b-3p | 0.001 | 0.000 |
| H19 | S1PR1 | hsa-miR-130a-3p,hsa-miR-454-3p,hsa-miR-130b-3p | 0.018 | 0.021 |
| H19 | TSPAN18 | hsa-miR-130a-3p,hsa-miR-454-3p,hsa-miR-130b-3p | 0.011 | 0.000 |
| H19 | CBX6 | hsa-miR-29c-3p,hsa-miR-29b-3p | 0.011 | 0.000 |
| H19 | AKT3 | hsa-miR-29c-3p,hsa-miR-29b-3p | 0.011 | 0.047 |
| H19 | CNTNAP1 | hsa-miR-370-3p | 0.025 | 0.000 |
| H19 | LOX | hsa-miR-29c-3p,hsa-miR-29b-3p | 0.014 | 0.004 |
| SNHG7 | GRIN2D | hsa-miR-342-3p | 0.022 | 0.008 |
| KCNQ1OT1 | SLC7A5 | hsa-miR-152-3p,hsa-miR-148a-3p | 0.005 | 0.000 |
| KCNQ1OT1 | CDC25B | hsa-miR-214-3p,hsa-miR-152-3p,hsa-miR-148a-3p | 0.021 | 0.001 |
| KCNQ1OT1 | ANKRD13B | hsa-miR-29c-3p,hsa-miR-29b-3p,hsa-miR-326,hsa-miR-330-5p | 0.006 | 0.000 |
| KCNQ1OT1 | NKRF | hsa-miR-29c-3p,hsa-miR-29b-3p,hsa-miR-377-3p | 0.021 | 0.000 |
| KCNQ1OT1 | SMYD5 | hsa-miR-214-3p | 0.041 | 0.001 |
| KCNQ1OT1 | COL7A1 | hsa-miR-29c-3p,hsa-miR-29b-3p | 0.009 | 0.001 |
| KCNQ1OT1 | MYBL2 | hsa-miR-29c-3p,hsa-miR-29b-3p | 0.021 | 0.002 |
| KCNQ1OT1 | TMEM97 | hsa-miR-214-3p,hsa-miR-7-5p | 0.000 | 0.019 |
| KCNQ1OT1 | SLC12A8 | hsa-miR-29c-3p,hsa-miR-29b-3p | 0.001 | 0.025 |
| KCNQ1OT1 | HELLS | hsa-miR-7-5p,hsa-miR-140-5p,hsa-miR-24-3p | 0.002 | 0.000 |
| KCNQ1OT1 | TRAF5 | hsa-miR-29c-3p,hsa-miR-29b-3p | 0.001 | 0.000 |
| KCNQ1OT1 | STX1A | hsa-miR-29c-3p,hsa-miR-29b-3p | 0.030 | 0.000 |
| KCNQ1OT1 | LMNB2 | hsa-miR-326,hsa-miR-24-3p,hsa-miR-330-5p | 0.002 | 0.006 |
| KCNQ1OT1 | SCML1 | hsa-miR-29c-3p,hsa-miR-29b-3p,hsa-miR-24-3p | 0.005 | 0.000 |
| KCNQ1OT1 | MEST | hsa-miR-29c-3p,hsa-miR-29b-3p | 0.001 | 0.004 |
| KCNQ1OT1 | ARHGEF19 | hsa-miR-29c-3p,hsa-miR-29b-3p | 0.001 | 0.000 |
| KCNQ1OT1 | SPRY4 | hsa-miR-29c-3p,hsa-miR-29b-3p,hsa-miR-140-5p | 0.027 | 0.019 |
| KCNQ1OT1 | POLR1D | hsa-miR-29c-3p,hsa-miR-29b-3p | 0.030 | 0.047 |
| KCNQ1OT1 | GGCT | hsa-miR-29c-3p,hsa-miR-29b-3p | 0.001 | 0.001 |
| KCNQ1OT1 | CDON | hsa-miR-377-3p,hsa-miR-152-3p,hsa-miR-148a-3p | 0.042 | 0.000 |
| KCNQ1OT1 | SLC2A1 | hsa-miR-328-3p,hsa-miR-140-5p,hsa-miR-152-3p,hsa-miR-148a-3p | 0.043 | 0.008 |
| KCNQ1OT1 | ABCB6 | hsa-miR-29c-3p,hsa-miR-29b-3p | 0.001 | 0.000 |
| KCNQ1OT1 | ARVCF | hsa-miR-29c-3p,hsa-miR-29b-3p | 0.002 | 0.000 |
| KCNQ1OT1 | ABLIM2 | hsa-miR-24-3p,hsa-miR-145-5p | 0.007 | 0.012 |
| HAGLR | EPB41L3 | hsa-miR-125a-3p | 0.047 | 0.001 |
| HAGLR | CAV1 | hsa-miR-125a-3p | 0.022 | 0.010 |
| MCF2L-AS1 | NUTF2 | hsa-miR-33a-5p | 0.018 | 0.000 |
| LINC00894 | GRIN2D | hsa-miR-342-3p | 0.022 | 0.000 |
| MAGI2-AS3 | SLC39A10 | hsa-miR-374b-5p,hsa-miR-374a-5p | 0.030 | 0.017 |
| MAGI2-AS3 | CDC14A | hsa-miR-374b-5p,hsa-miR-374a-5p | 0.007 | 0.000 |
| MAGI2-AS3 | MYLK | hsa-miR-374b-5p,hsa-miR-374a-5p | 0.005 | 0.000 |
| MAGI2-AS3 | PLPP3 | hsa-miR-374b-5p,hsa-miR-374a-5p | 0.016 | 0.000 |
| MAGI2-AS3 | NTN1 | hsa-miR-374b-5p,hsa-miR-374a-5p | 0.003 | 0.000 |
| MAGI2-AS3 | VAMP2 | hsa-miR-374b-5p,hsa-miR-374a-5p | 0.003 | 0.001 |
| MAGI2-AS3 | ZSWIM6 | hsa-miR-374b-5p,hsa-miR-374a-5p | 0.026 | 0.000 |
| MAGI2-AS3 | LPAR1 | hsa-miR-374b-5p,hsa-miR-374a-5p | 0.000 | 0.000 |
| MAGI2-AS3 | EDIL3 | hsa-miR-374b-5p,hsa-miR-374a-5p | 0.000 | 0.000 |
| MAGI2-AS3 | WASL | hsa-miR-374b-5p,hsa-miR-374a-5p | 0.049 | 0.000 |
| MAGI2-AS3 | ADAMTSL3 | hsa-miR-374b-5p,hsa-miR-374a-5p | 0.001 | 0.000 |
| MAGI2-AS3 | ATAD2 | hsa-miR-374b-5p,hsa-miR-374a-5p | 0.008 | 0.003 |
| MAGI2-AS3 | HOMER1 | hsa-miR-374b-5p,hsa-miR-374a-5p | 0.012 | 0.012 |
| MAGI2-AS3 | TNS1 | hsa-miR-374b-5p,hsa-miR-374a-5p | 0.011 | 0.000 |
| MAGI2-AS3 | MGAT4A | hsa-miR-374b-5p,hsa-miR-374a-5p | 0.031 | 0.000 |
| MAGI2-AS3 | NR3C1 | hsa-miR-374b-5p,hsa-miR-374a-5p | 0.032 | 0.000 |
| MAGI2-AS3 | CYBRD1 | hsa-miR-374b-5p,hsa-miR-374a-5p | 0.013 | 0.000 |
| MAGI2-AS3 | PDE4D | hsa-miR-374b-5p,hsa-miR-374a-5p | 0.032 | 0.000 |
| MAGI2-AS3 | DMD | hsa-miR-374b-5p,hsa-miR-374a-5p | 0.018 | 0.000 |
| MAGI2-AS3 | HSPH1 | hsa-miR-374b-5p,hsa-miR-374a-5p | 0.001 | 0.001 |
| MAGI2-AS3 | MEIS1 | hsa-miR-374b-5p,hsa-miR-374a-5p | 0.005 | 0.000 |
| MAGI2-AS3 | RECK | hsa-miR-374b-5p,hsa-miR-374a-5p | 0.036 | 0.000 |
| MAGI2-AS3 | PPP1R3C | hsa-miR-374b-5p,hsa-miR-374a-5p | 0.000 | 0.000 |
| MAGI2-AS3 | FGFR2 | hsa-miR-374b-5p,hsa-miR-374a-5p | 0.002 | 0.004 |
| MAGI2-AS3 | PMEPA1 | hsa-miR-374b-5p,hsa-miR-374a-5p | 0.006 | 0.000 |
| MAGI2-AS3 | ITGA2 | hsa-miR-374b-5p,hsa-miR-374a-5p | 0.007 | 0.005 |
| MAGI2-AS3 | ETNK1 | hsa-miR-374b-5p,hsa-miR-374a-5p | 0.042 | 0.011 |
| MAGI2-AS3 | DST | hsa-miR-374b-5p,hsa-miR-374a-5p | 0.001 | 0.000 |
| MAGI2-AS3 | ZEB2 | hsa-miR-374b-5p,hsa-miR-374a-5p | 0.030 | 0.000 |
| MAGI2-AS3 | BHLHE40 | hsa-miR-374b-5p,hsa-miR-374a-5p | 0.007 | 0.029 |
| MAGI2-AS3 | APC | hsa-miR-374b-5p,hsa-miR-374a-5p | 0.005 | 0.000 |
| MAGI2-AS3 | DIP2C | hsa-miR-374b-5p,hsa-miR-374a-5p | 0.012 | 0.000 |
| MAGI2-AS3 | TBC1D9 | hsa-miR-374b-5p,hsa-miR-374a-5p | 0.012 | 0.000 |
| MAGI2-AS3 | CDC42EP3 | hsa-miR-374b-5p,hsa-miR-374a-5p | 0.001 | 0.000 |
| MAGI2-AS3 | FAM46A | hsa-miR-374b-5p,hsa-miR-374a-5p | 0.035 | 0.007 |
| MAGI2-AS3 | SGPP1 | hsa-miR-374b-5p,hsa-miR-374a-5p | 0.010 | 0.000 |
| MAGI2-AS3 | WNT5A | hsa-miR-374b-5p,hsa-miR-374a-5p | 0.004 | 0.000 |
| MAGI2-AS3 | ANKRD12 | hsa-miR-374b-5p,hsa-miR-374a-5p | 0.014 | 0.000 |
| MAGI2-AS3 | PCDH7 | hsa-miR-374b-5p,hsa-miR-374a-5p | 0.010 | 0.000 |
| MAGI2-AS3 | TLE4 | hsa-miR-374b-5p,hsa-miR-374a-5p | 0.031 | 0.000 |
| MAGI2-AS3 | TTLL7 | hsa-miR-374b-5p,hsa-miR-374a-5p | 0.004 | 0.000 |
| MAGI2-AS3 | RIPOR2 | hsa-miR-374b-5p,hsa-miR-374a-5p | 0.000 | 0.000 |
| MAGI2-AS3 | ZCCHC24 | hsa-miR-374b-5p,hsa-miR-374a-5p | 0.013 | 0.000 |
| MAGI2-AS3 | ADGRL2 | hsa-miR-374b-5p,hsa-miR-374a-5p | 0.016 | 0.000 |
| MAGI2-AS3 | AKT3 | hsa-miR-374b-5p,hsa-miR-374a-5p | 0.016 | 0.000 |
| MAGI2-AS3 | ATP8B2 | hsa-miR-374b-5p,hsa-miR-374a-5p | 0.002 | 0.000 |
| MAGI2-AS3 | UST | hsa-miR-374b-5p,hsa-miR-374a-5p | 0.002 | 0.000 |
| MAGI2-AS3 | PI15 | hsa-miR-374b-5p,hsa-miR-374a-5p | 0.001 | 0.000 |
| MAGI2-AS3 | BTBD11 | hsa-miR-374b-5p,hsa-miR-374a-5p | 0.001 | 0.009 |
| MAGI2-AS3 | PDE10A | hsa-miR-374b-5p,hsa-miR-374a-5p | 0.005 | 0.000 |
| MAGI2-AS3 | L1CAM | hsa-miR-374b-5p,hsa-miR-374a-5p | 0.002 | 0.000 |
| MALAT1 | TARBP1 | hsa-miR-1271-5p,hsa-miR-96-5p | 0.002 | 0.005 |
| AC015813.1 | RPGRIP1L | hsa-miR-590-3p | 0.022 | 0.000 |
| AC015813.1 | NOLC1 | hsa-miR-590-3p | 0.011 | 0.000 |
| AC015813.1 | PPA1 | hsa-miR-590-3p | 0.004 | 0.028 |
| AC015813.1 | DSN1 | hsa-miR-590-3p | 0.007 | 0.000 |
| AC015813.1 | SUV39H2 | hsa-miR-590-3p | 0.018 | 0.000 |
| AC015813.1 | PABPC1 | hsa-miR-590-3p | 0.022 | 0.015 |
| AC015813.1 | ARVCF | hsa-miR-590-3p | 0.014 | 0.000 |
| AC015813.1 | SERPINE2 | hsa-miR-590-3p | 0.025 | 0.023 |

ceRNAs: competing endogenous RNAs; LncRNA: long non-coding RNA; MiRNA: microRNA.
